# Revolutionizing stroke care in Africa: A mini review of the transformative potential of mobile stroke units

**DOI:** 10.1097/MD.0000000000035899

**Published:** 2023-11-03

**Authors:** Gbolahan Olatunji, Emmanuel Kokori, Timilehin Isarinade, Ismail Yusuf, Chidinma I. Udojike, Oluwaseun Abimbola, Samuel Owolabi, Muili Opeyemi Abdulbasit, Nicholas Aderinto

**Affiliations:** a Department of Medicine and Surgery, University of Ilorin, Ilorin, Nigeria; b Department of Medicine and Surgery, Obafemi Awolowo University, Ile-Ife, Nigeria; c Department of Medicine and Surgery, University of Lagos, Lagos, Nigeria; d Lagos State Health Services Commission, Lagos, Nigeria; e Department of Medicine and Surgery, Ladoke Akintola University Teaching Hospital, Lagos, Nigeria.

**Keywords:** Africa, healthcare, mobile stroke units, stroke care

## Abstract

Stroke is a major health concern worldwide, and its impact is particularly pronounced across Africa. This paper delves into the challenges faced in African stroke care and explores the significant potential benefits of mobile stroke units (MSUs) in mitigating these issues. Key challenges include the limited healthcare infrastructure, funding constraints, difficulties reaching remote and rural areas, and shortages of qualified healthcare professionals, especially neurologists and stroke specialists. To address these challenges, recommendations are provided, emphasizing the importance of infrastructure development, sustainable funding mechanisms, solutions for rural accessibility, and healthcare workforce development through training programs and incentives. Additionally, the paper discusses prospects for MSUs in Africa, highlighting the potential for technology advancements to yield more cost-effective and compact MSU models. The integration of telemedicine capabilities within MSUs is examined to enhance communication with specialist physicians at remote hospitals, ultimately improving stroke care outcomes. Furthermore, data collection on MSU outcomes and their impact on stroke care is emphasized to inform evidence-based policies and enhance MSU operations. Collaboration and partnerships between governments, healthcare organizations, and international stakeholders are critical for facilitating MSU expansion. These partnerships can provide essential funding, expertise, and support for the implementation and sustainable operation of MSUs in Africa.

## 1. Introduction

Every second counts when managing a stroke.^[1]^ As the saying goes, “Time is Brain.”^[1]^ This phrase underscores the critical importance of swift intervention in stroke management. However, achieving timely stroke care presents formidable challenges, especially in developing countries like those in Africa. These challenges compound delays in stroke care, stemming from suboptimal healthcare-seeking behavior, inadequate road infrastructure, and a shortage of specialized facilities.^[2,3]^ Recent data from Africa reveal alarming statistics: an annual stroke incidence rate of up to 316 per 100,000, a prevalence as high as 1460 per 100,000, and a 3-year fatality rate exceeding 80%.^[4]^ In Africa, where stroke mortality and disability rates remain elevated due to limited access to specialized facilities and delayed treatment, the urgency for innovative solutions cannot be overstated.

The mobile stroke unit (MSU) concept was first introduced in 2003.^[5]^ An MSU is a highly specialized vehicle equipped with cutting-edge medical technology to provide immediate care to hyperacute stroke patients before they reach the hospital. These units typically feature a CT scanner, telemedicine capabilities, a multidisciplinary medical team, enhanced communication with emergency services, and basic laboratory facilities.^[6]^ The deployment of the first MSUs in Germany in 2010 and 2011 marked a transformative moment in stroke care.^[7]^ After the United States embraced the concept and deployed its inaugural MSU in 2014, it rapidly gained nationwide traction.^[7]^ One of the most remarkable attributes of MSUs is their ability to significantly shrink the time between the onset of stroke symptoms and the commencement of treatment.^[7]^ This critical time window, often called the “golden hour,” is pivotal in stroke care. Interventions like thrombolysis are most effective during this crucial window, substantially enhancing the chances of a successful outcome.^[8,9]^ These specialized vehicles are designed to bring stroke care directly to the patient’s doorstep, effectively bridging the geographical and logistical barriers that hinder timely treatment.

Research has consistently demonstrated that MSUs drastically reduce the time from symptom onset to the initiation of treatment. For instance, in Germany, where MSUs were first introduced, studies have reported a median reduction in treatment time of approximately 25 minutes for stroke patients transported by MSUs compared to traditional ambulances.^[8]^ Reducing treatment time is pivotal in curbing stroke morbidity and mortality.^[9]^ By expediting care delivery, MSUs enhance the prospects of positive outcomes and reduce the risk of long-term disability. Similar encouraging outcomes have been observed in the United States, where MSUs have slashed treatment times and increased the rate of thrombolysis administration, a critical factor in improving stroke outcomes.^[10]^ This paper underscores the acute need for enhanced stroke care in Africa, where high mortality and disability rates are perpetuated by limited access to specialized facilities and delays in treatment. By examining the transformative potential of MSUs, the paper explores the possibilities of revolutionizing stroke care in the African context.

## 2. Methodology

To comprehensively explore the transformative potential of MSUs in revolutionizing stroke care in Africa, we conducted a narrative review. We initiated the study by conducting a literature search across various databases, including PubMed, Google Scholar, Scopus, Web of Science, and African Journals Online (AJOL). We sought articles published between 2000 and 2023 to encompass the most recent developments in the field. The search was guided by a set of keywords related to “mobile stroke units,” “stroke care,” “Africa,” and associated terms. Boolean operators were used to refine search results, and we considered both peer-reviewed journal articles and gray literature.

Articles were included if they directly addressed the utilization and potential impact of MSUs on stroke care in Africa, discussed the challenges and opportunities for improving stroke care in African settings, were published within our specified timeframe, and were available in English or had an English translation. Conversely, we excluded studies unrelated to MSUs or stroke care in Africa, lacked essential information or displayed methodological flaws, and instances of duplicate publications. The data were analyzed thematically, allowing us to identify key findings, trends, and gaps in the existing literature.

### 2.1. Current challenges in stroke care in Africa

Stroke care in Africa faces many challenges that undermine the quality and effectiveness of stroke management. These challenges are deeply rooted in many African nations’ economic, social, and healthcare disparities. In understanding these challenges, it becomes evident that addressing them is not only a matter of healthcare but also a crucial component of broader efforts to improve public health, strengthen healthcare systems, and reduce the burden of noncommunicable diseases in Africa.

#### 2.1.1. Healthcare infrastructure and access.

Africa’s vast and diverse geography poses significant challenges in delivering stroke care.^[1]^ Access to well-equipped healthcare facilities is limited in many rural and remote areas, and specialized stroke units are often nonexistent.^[2]^ The scarcity of essential diagnostic equipment, such as CT scanners and MRI machines, further compounds the problem. Patients from these regions face substantial delays in receiving care due to the long distances they must travel to reach healthcare facilities. Moreover, the state of road infrastructure often impedes transportation, leading to life-threatening delays.^[3]^

#### 2.1.2. Human resources and expertise.

The shortage of skilled healthcare personnel, particularly neurologists and specialists in stroke care, is a pressing issue in Africa. The World Health Organization recommends a minimum ratio of one neurologist per 50,000 people for adequate stroke care.^[3]^ However, many African countries fall far short of this benchmark. In some nations, there are as few as one neurologist for every 500,000 people.^[4]^ This scarcity limits the availability of specialized stroke care and expertise, contributing to delayed diagnosis and treatment.

#### 2.1.3. Financial barriers and healthcare funding.

Financial barriers loom large in providing African stroke care. Inadequate healthcare funding is a pervasive issue, with many nations failing to allocate the recommended 15% of their budgets to the health sector, as outlined in the Abuja Declaration of 2001.^[5]^ This funding shortfall leads to underfunded healthcare facilities, a lack of essential resources, and compromised stroke care services. Patients and their families often bear the financial burden, as health insurance systems are frequently underdeveloped or inaccessible.^[6]^ Out-of-pocket expenses for stroke care, including diagnostic tests and medications, can become prohibitively expensive, further deterring timely care.

#### 2.1.4. Public awareness and education.

A fundamental issue is the low level of awareness among the general population regarding stroke symptoms, risk factors, and the urgency of seeking immediate medical attention.^[7]^ This lack of awareness leads to delayed presentation to healthcare facilities and contributes to inappropriate responses when individuals experience stroke symptoms.^[8,9]^

### 2.2. The innovation of MSUs

In 2008, a groundbreaking approach to stroke care emerged with the introduction of MSUs.^[10]^ These specialized vehicles have rapidly gained recognition as a transformative solution for managing strokes before patients reach the hospital. While conventional stroke care has traditionally revolved around transporting patients to the nearest stroke-ready hospital, providing critical care directly at the emergency site was initially proposed in 2003 and effectively implemented in 2008.^[1]^

**Figure 1.**
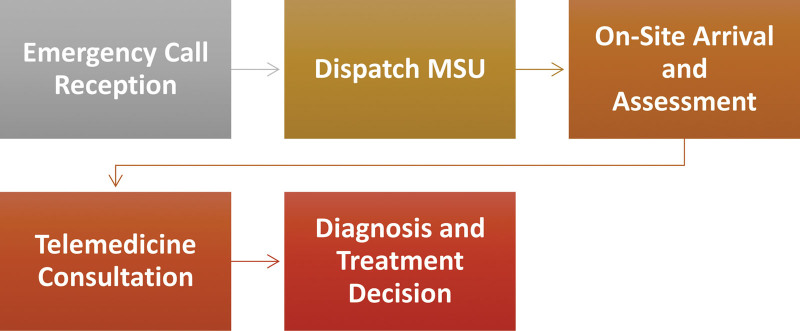
Mobile stroke unit workflow.

The essence of MSUs revolves around a comprehensive approach to stroke care.^[2]^ It encompasses a meticulous evaluation of the patient’s medical history conducting clinical and neurological assessments, all under the guidance of a specialized team armed with diagnostic and treatment tools^[3]^ Figure 1. These tools include cutting-edge CT scanners, point-of-care (POC) laboratory facilities, telemedicine capabilities, and a cache of advanced stroke medications within a specially equipped ambulance.^[4]^ This setup empowers medical professionals to make pivotal decisions regarding thrombolysis interventions immediately, dramatically reducing the time gap between the onset of stroke symptoms and the initiation of treatment.^[5]^

One of the most significant advantages of MSUs is their capacity for diagnosis-based triage.^[6]^ This entails determining the most appropriate target hospital for each patient’s condition. MSUs work with hospital-based stroke units, extending specialized care into the crucial prehospital phase.^[7]^

The design of MSU ambulances is globally variable and tailored to meet regional needs and demands.^[8]^ Some prioritize smaller, lightweight solutions, ensuring agility and maneuverability, particularly useful for navigating narrow roads.^[9]^ In contrast, others opt for larger vehicles capable of accommodating additional equipment and even the patient’s family members.^[10]^

Central to the functionality of MSUs is cerebral imaging, a pivotal component in assessing stroke cases.^[1]^ It serves a dual purpose: determining a patient’s eligibility for tissue plasminogen activator administration and assessing whether the patient requires transfer to a comprehensive stroke center for intra-arterial treatment. To address the challenge of inconsistent assessments of CT scans, automated image analysis tools have been developed.^[2]^ These tools contribute to standardized scan evaluations, aiding in determining the suitability for thrombolysis and detecting large vessel occlusion.^[3]^

MSUs are equipped with POC laboratories capable of conducting various tests, including hematological, clinical chemistry, and coagulation marker tests.^[4]^ This capability enables the swift assessment of renal function, a crucial factor for CT angiography procedures.^[5]^ Effective communication between MSUs and hospitals is paramount, involving live two-way audio-visual communication and real-time sharing of patient data and CT scans.^[6]^ This telemedicine capacity empowers the MSU team to receive expert guidance from the hospital. Regarding staffing and operation, MSUs can function independently and in collaboration with traditional Emergency Medical Services units, a flexibility contingent on regional regulations and available resources.^[7]^ The hallmark of MSUs lies in their highly organized teamwork, minimizing the time elapsed from the onset of stroke symptoms to the initiation of treatment.

### 2.3. Potential benefits of MSUs for stroke care in Africa

**Figure 2.**
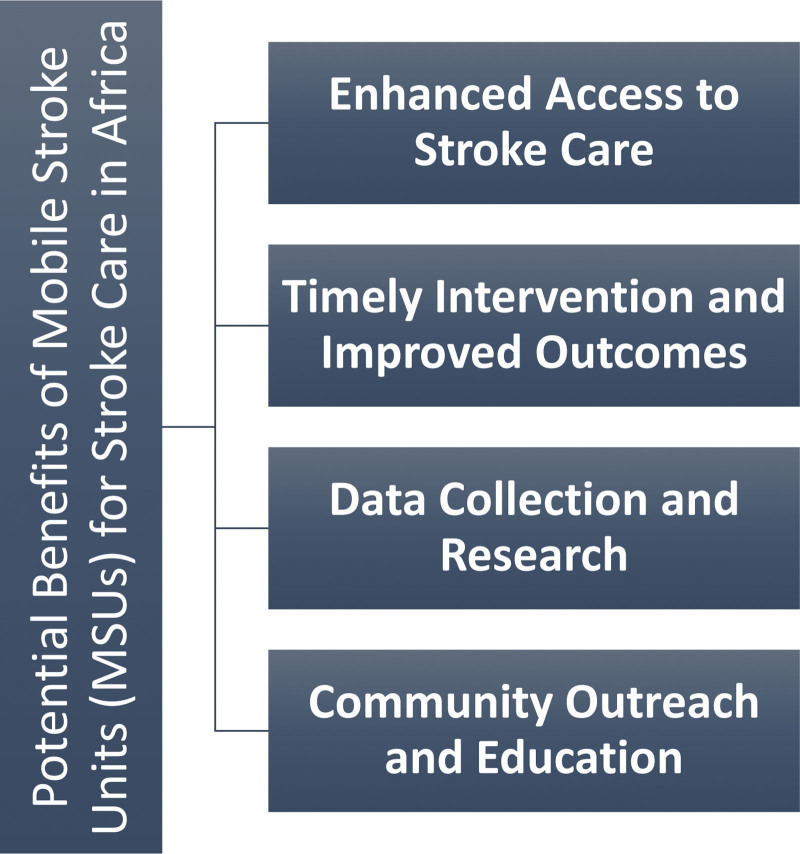
Potential benefits of mobile stroke units (MSUs) for stroke care in Africa.

MSUs offer substantial potential benefits for stroke care in Africa, which faces unique challenges in timely and effective stroke management—Figure 2.

#### 2.3.1. Enhanced access to stroke care.

MSUs have the potential to revolutionize access to stroke care in Africa, particularly in remote and underserved areas. Geographical barriers, limited healthcare infrastructure, and inadequate road networks have historically hindered the delivery of specialized stroke care to rural populations.^[2]^ However, MSUs can address these challenges effectively. In regions where traditional ambulance services struggle to reach due to difficult terrain, MSUs can navigate challenging routes, ensuring that individuals living in remote or inaccessible areas receive timely medical attention.^[1]^ This not only reduces transport time but also extends stroke care services to populations that were previously underserved. Furthermore, MSUs are crucial in addressing healthcare disparities between urban and rural areas.^[2]^ By bringing advanced medical services closer to remote communities, they reduce inequality in stroke care outcomes. This enhanced access ensures that stroke patients, regardless of geographic location, have a better chance of receiving life-saving interventions.

#### 2.3.2. Timely intervention and improved outcomes.

One of the fundamental principles of stroke care is that “time is brain.^[1]^” MSUs embody this principle by providing rapid on-site stroke care.^[10]^ By delivering critical medical interventions directly at the emergency site, MSUs significantly reduce the time between the onset of stroke symptoms and the initiation of treatment.^[3]^ The concept of the “golden hour,” which represents the critical period for optimal stroke intervention, is maximized through MSUs.^[1]^ These units ensure patients receive assessment, diagnosis, and treatment during this crucial window. As a result, there is a potential for reduced disability and mortality rates among stroke patients. Additionally, MSUs streamline the care workflow. Medical professionals aboard these units can swiftly assess the patient’s condition, make informed decisions, and initiate appropriate interventions.^[4]^ This approach results in more efficient and effective care delivery, ultimately improving patient outcomes.

#### 2.3.3. Data collection and research.

MSUs offer an opportunity to collect valuable data on stroke incidence and care quality in Africa. This data is instrumental in understanding the prevalence of strokes, identifying high-risk areas, and tailoring healthcare strategies accordingly.^[5]^ Real-time data collection on patient outcomes and the effectiveness of prehospital stroke care is another significant benefit. MSUs provide a platform for gathering data on treatment responses, recovery rates, and long-term outcomes. This data-driven approach informs healthcare providers and policymakers about the impact of MSUs on stroke care quality.^[6]^ Moreover, the presence of MSUs encourages research and analysis of stroke-related trends. Researchers can study patient demographics, treatment protocols, and the influence of various factors on stroke care. These insights contribute to the continuous improvement of stroke care protocols, ensuring that they align with the unique needs of African populations.

#### 2.3.4. Community outreach and education.

MSUs extend their impact beyond immediate medical care by engaging with communities and promoting stroke awareness and education. These units are pivotal in raising awareness about stroke symptoms, risk factors, and the critical importance of early intervention.^[7]^ Community education programs conducted during MSU visits empower individuals to recognize the signs of stroke and understand the urgency of seeking immediate medical help.^[8]^ By disseminating this knowledge, MSUs contribute to reducing the time it takes for individuals to react to stroke symptoms, thereby enhancing the chances of timely treatment. Furthermore, MSUs help build trust and engagement between healthcare providers and communities.^[9]^ By delivering care directly to people’s doorsteps, these units foster stronger connections between medical professionals and the population they serve. This trust is essential in encouraging individuals to seek prompt medical assistance for stroke, ultimately improving outcomes.

### 2.4. Challenges and recommendations

MSUs have emerged as a transformative approach to stroke care globally. In Africa, MSUs hold the potential to significantly reduce the devastating impact of strokes by providing rapid, on-the-spot diagnosis and treatment. However, while MSUs have shown remarkable success in various regions, their adoption in Africa presents unique challenges and opportunities Table 1.

**Table 1:** Challenges and recommendations for implementing mobile stroke units (MSUs) in Africa.

Challenges	Recommendations
Infrastructure and resource limitations	-Prioritize investments in healthcare infrastructure, including road network improvements for better MSU access. -Procure essential medical equipment and diagnostic tools suitable for MSUs. -Focus on acquiring mobile and compact devices for efficiency.
Funding and sustainability	-Secure sustainable funding through public–private partnerships, health insurance schemes, and international aid. -Allocate a dedicated budget for MSU operation and maintenance. -Explore collaborations with NGOs and international healthcare agencies for financial support.
Rural accessibility	-Integrate telemedicine solutions to connect MSUs with remote healthcare centers. -Enable real-time consultations with specialists to facilitate decision-making in areas with limited physical access.
Healthcare workforce	-Invest in comprehensive training programs for neurologists, stroke specialists, and paramedics to address shortages. -Offer scholarships and incentives to healthcare professionals to work in underserved areas. -Collaborate with universities and medical institutions to facilitate specialized training for MSU teams.

#### 2.4.1. Infrastructure and resource limitations.

One of the foremost challenges in implementing MSUs for stroke care in Africa is the inadequacy of healthcare infrastructure and resources.^[10]^ Many regions grapple with limited, often poorly maintained road networks, rendering swift access to remote areas a formidable task.^[1]^ Additionally, the scarcity of essential medical equipment, including specialized CT scanners and POC laboratories, coupled with a shortage of qualified medical personnel, can lead to substantial delays in responding to stroke emergencies, particularly in remote and underserved regions.^[2]^ To overcome these infrastructure limitations, governments and healthcare authorities must prioritize investments in healthcare infrastructure. This includes improving road networks and ensuring MSUs can access remote areas efficiently. Furthermore, procuring essential medical equipment and diagnostic tools should be a priority, focusing on acquiring mobile and compact devices suitable for MSUs.

#### 2.4.2. Funding and sustainability.

Establishing and maintaining MSUs necessitate substantial financial investments.^[3]^ Acquiring the initial funds for purchasing specialized vehicles, medical equipment, and staff training poses a formidable challenge. Equally vital is securing sustainable funding to ensure the uninterrupted functioning of MSUs. Budget constraints within African healthcare systems can jeopardize the long-term viability of these units, potentially leading to service interruptions and compromised stroke care. Achieving sustainable funding for MSUs can be realized through public and private partnerships, health insurance schemes, and international aid. Governments should allocate a dedicated budget for the operation and maintenance of MSUs and explore collaborations with nongovernmental organizations and international healthcare agencies to provide crucial financial support.

#### 2.4.3. Rural accessibility.

Providing equitable access to stroke care in remote and rural areas is a considerable challenge.^[4]^ Geographical barriers, such as rugged terrain and vast distances, often impede the timely arrival of MSUs in these areas. Poor road conditions and a dearth of transportation options exacerbate the problem, making it difficult for these units to reach needy patients. To enhance rural accessibility, the integration of telemedicine solutions is highly recommended. MSUs can employ telemedicine to connect with remote healthcare centers and consult with specialists in real time. This approach can facilitate rapid decision-making and treatment planning, even in areas with limited physical access.

#### 2.4.4. Healthcare workforce.

The shortage of qualified healthcare professionals, particularly neurologists, and stroke specialists, presents a significant workforce challenge.^[2]^ Operating MSUs demands a skilled and trained team capable of making rapid, accurate diagnoses and administering timely treatment.^[5]^ However, in many African countries, the availability of specialized stroke care training programs is limited. Furthermore, recruiting and retaining healthcare professionals in underserved areas can be arduous, often requiring incentives and support systems. Effective multidisciplinary collaboration is pivotal, especially in regions where healthcare personnel are scarce, to ensure the seamless functioning of MSUs and the delivery of quality stroke care. Governments should invest in comprehensive training programs for neurologists, stroke specialists, and paramedics to address the healthcare workforce challenges. Scholarships and incentives can encourage healthcare professionals to work in underserved areas, while collaborations with universities and medical institutions can facilitate specialized training for MSU teams.

## 3. Future prospects for MSUs in stroke in Africa

The future of MSUs in Africa is marked by tremendous potential for transformation and innovation in stroke care. Technology advancements, the integration of telemedicine, the importance of research and data, and the power of collaboration and partnerships will shape the trajectory of MSUs in the African healthcare landscape. These elements collectively promise to deliver more accessible, efficient, and impactful stroke care to communities across the continent.

### 3.1. Technology advancements

The future of MSUs in Africa holds promise, driven by ongoing technological advancements. These innovations will likely lead to the developing of more cost-effective and compact MSU models. These streamlined and efficient units can make MSUs accessible to healthcare settings with limited resources, particularly in resource-constrained regions of Africa. Miniaturized diagnostic equipment, more energy-efficient ambulance designs, and optimized power sources can significantly contribute to the sustainability and scalability of MSUs.

### 3.2. Telemedicine integration

The integration of telemedicine capabilities within MSUs represents a pivotal advancement. This integration has the potential to transform stroke care in Africa. By incorporating telemedicine, MSUs can establish real-time communication with specialist physicians at remote hospitals. This connection allows for immediate consultations, guidance, and expertise exchange. Through telemedicine, even the most remote areas can benefit from the knowledge and experience of stroke specialists. Telemedicine can bridge the expertise gap and significantly improve stroke care outcomes, particularly in regions with limited access to specialized care.

### 3.3. Research and data

A key aspect of the future of MSUs in Africa is the collection of robust data on their outcomes and impact on stroke care. Research efforts should focus on systematically gathering data on MSU operations, patient outcomes, and treatment efficacy. This data-driven approach is essential for building a strong evidence base. The findings from such research can inform evidence-based policies and guidelines for stroke care. Additionally, research can shed light on areas of improvement and innovation, ensuring that MSUs continue to evolve to meet the specific needs of African healthcare systems.

### 3.4. Collaboration and partnerships

Collaborative efforts between governments, healthcare organizations, and international partners will play a crucial role in shaping the future of MSUs in Africa. Establishing partnerships with various stakeholders can provide the necessary resources, expertise, and support for the successful implementation and operation of MSUs. Governments should actively engage with international agencies, nongovernmental organizations, and philanthropic foundations to secure funding and technical assistance. These collaborations can facilitate the expansion of MSUs across African countries and regions, ensuring that more communities have access to timely and effective stroke care.

## 4. Conclusion

Adopting MSUs will represent a pioneering leap forward for stroke care in Africa. MSUs can potentially address the urgent need for timely intervention and the myriad challenges posed by Africa’s diverse and often resource-constrained healthcare settings. Challenges abound, from infrastructural limitations to funding constraints, rural accessibility, and workforce shortages. These hurdles, however, are not insurmountable. The recommendations outlined in this paper offer a strategic roadmap for policymakers, healthcare organizations, and stakeholders to navigate these challenges effectively. Through concerted efforts, Africa can strengthen its healthcare infrastructure, secure sustainable funding, enhance rural accessibility, and cultivate a skilled healthcare workforce, all of which are vital for the successful implementation and operation of MSUs.

## Author contributions

**Conceptualization:** Nicholas Aderinto.

**Writing – original draft:** Gbolahan Olatunji, Emmanuel Kokori, Timilehin Isarinade, Ismail Yusuf, Chidinma I. Udojike, Oluwaseun Abimbola, Samuel Owolabi, Muili Opeyemi Abdulbasit, Nicholas Aderinto.

**Writing – review & editing:** Gbolahan Olatunji, Emmanuel Kokori, Timilehin Isarinade, Ismail Yusuf, Chidinma I. Udojike, Oluwaseun Abimbola, Samuel Owolabi, Muili Opeyemi Abdulbasit, Nicholas Aderinto.
